# Object-Level Double Constrained Method for Land Cover Change Detection

**DOI:** 10.3390/s19010079

**Published:** 2018-12-26

**Authors:** Zhihao Wang, Yalan Liu, Yuhuan Ren, Haojie Ma

**Affiliations:** 1 Institute of Remote Sensing and Digital Earth, Chinese Academy of Sciences, Beijing 100101, China; wangzh2016@radi.ac.cn (Z.W.); renyuhuan_2001@163.com (Y.R.); mahj@radi.ac.cn (H.M.); 2 University of Chinese Academy of Sciences, Beijing 100101, China

**Keywords:** land cover change detection, object-level, multi-scale segmentation, optimal features selection, change vector analysis, correlation coefficient

## Abstract

Land cover change detection based on remote sensing has become increasingly important for protecting the ecological environment. Spatial features of images can be extracted by object-level methods. However, the computational complexity is high when using many features to detect land cover change. Meanwhile, single-constrained change detection (SCCD) methods produce non-objective and inaccurate results. Therefore, we proposed a land cover change detection method: the object-level double constrained change detection (ODCD) method. First, spectral and spatial features were calculated based on multi-scale segmentation results. Second, using the significant difference test (SDT), feature differences among all categories were calculated, and the features with more significant differences were considered as the optimal features. Third, the maximum Kappa coefficient was used as the criterion for determining the optimal change intensity and correlation coefficient. Finally, the ODCD was validated using GF-1 satellite images on March 2016 and February 2017 in north Beiqijia Town, Beijing. Using optimal feature selection, the dimension of features was reduced from 26 to 12. Compared with SCCD methods, the result of the ODCD was more reliable and accurate. Its overall accuracy was 10% higher, overall error was 27% lower, and the Kappa coefficient was 0.22 higher. In conclusion, the ODCD is effective for land cover change detection and can improve computational efficiency.

## 1. Introduction

Owing to the rapid increase of urban populations and the rapid expansion of urban areas, many ecological and environmental problems, such as reduced vegetation cover and increased surface runoff, have become gradually more serious [1]. As the core of ecological environment change monitoring, land cover change detection has become a hot topic in environmental science and ecology [2]. Remote sensing technology has the advantages of being macroscopic, comprehensive, dynamic, and rapid, as well as being the most economical and effective means for detecting land cover changes [3]. Various remote-sensing methods have been applied to this problem: Yuan et al. used Principal Component Analysis (PCA) to identify land cover changes based on multi-temporal Landsat5 TM images [4]; Johnson et al. used Change Vector Analysis (CVA) to detect land cover changes in Landsat5 TM multispectral images [5]; Zhou and Yang used ratios of different images to detect changes in Anshun City, Guizhou Province [6]; and Li and Ye used PCA to detect changes in Dongguan, Zhujiang Delta [7]. However, these methods were all on the pixel-level; thus, they cannot use the spatial characteristics of images and are prone to the serious “pepper and salt phenomenon” [8]. Due to improvements in spatial resolution, the remote sensing images have become increasingly informative. Im et al. first introduced the object-level method for land cover classification and change detection [9]. Lobo et al. compared object-level methods with pixel-level methods and found that the results from the former are more easily interpreted and have better integrity for each patch [10]. Wang and Zhao detected the land cover changes on the object-level using high-resolution remote sensing images [11]. Moreover, Lu and Scott used object-level methods to detect urbanization changes in high-resolution images [12]. These results showed that the object-level methods had more advantages than the pixel-level methods. However, objects generated by object-level segmentation have multi-dimensional features for remote sensing images, i.e., spectral, texture, and spatial features, and as the number of bands increases, the number of features also increases, which will increase the computational burden if all the features are used to perform land cover change detection. That is, as the dimension of the features increases to a certain number, the change detection accuracy decreases [13]. In addition, object-level change detection methods include a direct comparison method and a post-classification comparison method [14]. However, the object-level post-classification comparison method relies on the effect of classification, which has limitations, whilst the object-level direct comparison method has better stability and accuracy [15]. In recent decades, many scholars have continuously developed many object-level detection methods based on remote sensing. Quampby compared the differences of bands in images to detect urban land-use change [16]; Fan et al. used the image difference method to detect changes in Panzhihua [17]; and Li et al. used PCA to detect changes in Dongguan of the Pearl River Delta [18]. Yan proposed an object-level method. The author used the Mean-Shift algorithm to segment the image and used CVA to obtain the detection result [19]. However, most of these methods used one threshold to detect change, which rendered the results not objective. Object-level CVA is a direct comparison change detection method, which can usually obtain good results. For example, Yu used Landsat TM/ETM+ to compare the object-level CVA with the object-level conventional change detection methods (spectral vector similarity method, principal component difference method), and found that the object-level CVA obtained the best results of land cover change detection [20]. Qi et al. applied object-level CVA to perform land cover change detection with polarized SAR images, which improved the accuracy of change detection [21]. Wang et al. used object-level CVA for land cover change detection and they obtained good results [22]. At the same time, J. Im et al. studied the correlation between image objects based on segmentation and they extracted the change regions based on this method. The authors’ found that with the improvement in image resolution, the object-level correlation analysis was suitable for change information extraction of remote sensing images [9]. Given that CVA can directly compare the differences between image features and that the correlation coefficient can analyze the correlation between image features, the combination of the two methods can more effectively detect land cover change. However, as the number of bands increases, the judgement of change using CVA becomes relatively difficult [23,24]. Moreover, the change threshold is usually determined by empirical judgment that makes the results neither objective nor effective. Therefore, this study proposed a method of double constrained thresholds for the change intensity threshold and the correlation coefficient on the object-level (ODCD), which aimed to reduce the number of dimensions for features and to improve the computational efficiency, objectivity, and accuracy of land cover change detection.

## 2. Materials and Methods

### 2.1. Materials

**Figure 1:**
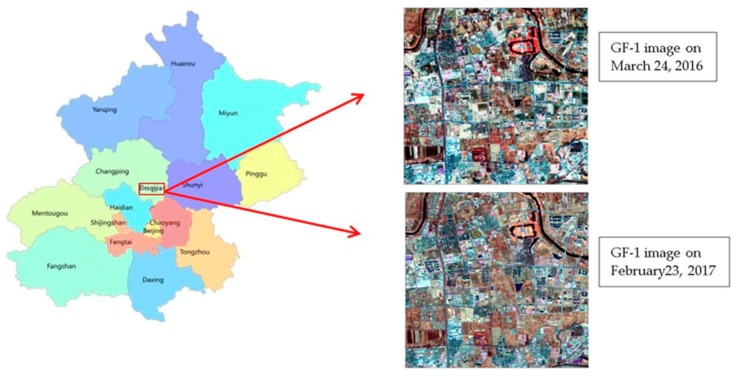
Map and GF-1 satellite images of the study area.

The study area is located at the northern part of Beiqijia Town, Changping, Beijing (Figure 1). The typical classes of land cover in this area are vegetation, residential, bare land, and waterbody. The data used in this study were 8-m resolution multi-spectral images of the GF-1 satellite acquired on 24 March 2016 and 23 February 2017.

GF-1 satellite is a high-resolution earth observation system remote sensing satellite launched by China on 26 April 2013. GF-1 satellite is equipped with two cameras (PMS) with a 2-m resolution panchromatic wave band, 8-m resolution multi-spectra band, and four 16-m resolution multi-spectral wide-format cameras (WFV1–WFV4). Its multispectral sensors have four bands: blue, green, red, and near infrared. The interview period is 4 days. GF-1 satellite data has the characteristics of high resolution, wide width, and short return period, and it can be widely used in agricultural remote sensing, environmental monitoring, and other fields [25,26].

### 2.2. Methods

This study used object-level CVA and the correlation coefficient to achieve land cover change detection. To reduce redundancy of data and to improve the quality of selected features, the significant difference test (SDT) for features was performed to select the most significant difference feature as the optimal feature. When determining the optimal change threshold, the optimal thresholds of change intensity and the correlation coefficient were selected based on the maximum Kappa coefficient. The Kappa coefficient is a consistency test method proposed by Cohen in 1960 to evaluate the classification results of remote sensing images [27,28]. It is calculated according to Equations (1) and (2).

(1) $$k=\frac{P_{0}-P_{e}}{1-P_{e}}$$

(2) $$P_{0}=\frac{\sum_{i=1}^{n}P_{ii}}{N}$$

where *k* is the Kappa coefficient. $P_{0}$ is the proportion of units in which the judges agreed; $P_{e}$ is the proportion of units for which agreement is expected by chance; *n* is the number of types of classification; *N* is the total number of samples; $P_{ii}$ is the number of correctly classified samples of type *i*. 

**Figure 2:**
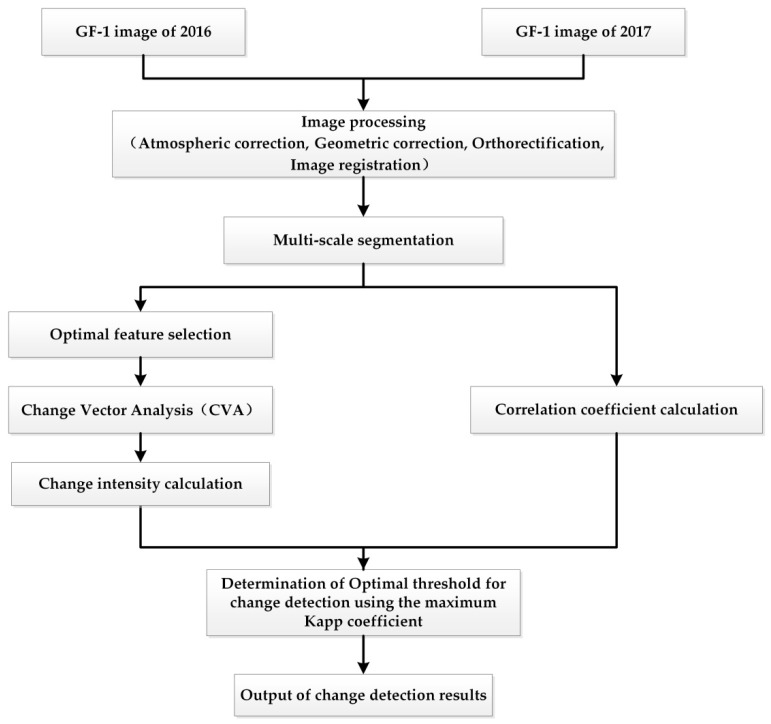
Flow chart of object-level double constrained change detection (ODCD).

The flow chart for the ODCD is shown in Figure 2, which includes the following steps. First, the atmospheric correction, geometric correction, orthorectification, and image registration were performed to reduce noises and improve image quality. Second, multi-scale segmentation was used to obtain highly homogeneous objects, and the initial land cover categories, such as classification of vegetation, bare land, residential, and waterbody. Third, some common spectral features, shape features, and texture features of objects were calculated based on the multi-scale segmentation results. Fourth, using SDT, the differences of the above features amongst all the categories were calculated, and the features with more significant differences were considered as the optimal features. Fifth, the change intensity was calculated via CVA using the optimal features, and the correlation coefficient between corresponding objects in the GF-1 image of 2016 and 2017 were calculated. Sixth, based on the change intensity and the correlation coefficient calculated in the previous step, the maximum Kappa coefficient was used as the criterion for determining the optimal thresholds of the change intensity and the correlation coefficient. Seventh, using the optimal thresholds of the change intensity and the correlation coefficient, the results of land cover change detection could be obtained. Finally, for the overall accuracy, the Kappa coefficient and the overall error were selected as the accuracy evaluation indexes. 

#### 2.2.1. Multi-Scale Segmentation

Multi-scale segmentation is a widely used image segmentation method that exhibits good results for object-level remote sensing image analysis. It comprehensively considers the spectral features and spatial features of remote sensing images, and it uses a bottom-up iterative merging algorithm to segment an image into objects with high homogeneity [29]. The homogeneity of objects is calculated as the standard deviation of the objects’ internal pixels, whilst the heterogeneity includes the spectral and shape heterogeneity of objects [30]. In this study, both images underwent multi-scale segmentation simultaneously to obtain the same segmentation objects. This ensured that the homogeneous objects did not include multiple objects or mixed objects in the other image, if only a single image was used for segmentation. Furthermore, the segmentation results should not be too fragmented and the differences between neighboring objects are demonstrated by setting the segmentation scale. The objects should have high internal homogeneity and be consistent with the actual boundaries of features. The main parameters of multi-scale segmentation include the segmentation scale and homogeneity factor. The homogeneity factor includes spectral features and shape factors, and the sum of the weights of the spectral features and shape factors is 1. Spectral features are typically the most important, being important factors for image object generation. When the weight of the shape factor is higher than 0.5, the generated polygons are too regular and have no practical meaning, and therefore, do not conform to the actual features of the objects [31]. Thus, the weight of the spectral features should be greater than 0.6 [32,33].

#### 2.2.2. Optimal Feature Selection

##### Feature Construction

Object-level change detection can not only utilize the spectral features, but also the spatial features of images, including texture features and shape features, to obtain more descriptive information. In traditional change detection methods, spectral features are the most important factor due to visual expression of image information [34]. In this study, four commonly used spectral features, including the mean, standard deviation, normalized difference vegetation index (NDVI), and the normalized difference water index (NDWI) were selected, and the features’ calculation formulas are shown in Table 1.

Texture features can reflect the regional characteristics of a remote sensing image. In 1973, Haralick proposed the characteristic parameters for analyzing the Gray Level Co-occurrence Matrix (GLCM) [35]. The GLCM is a widely used method for calculating texture features. In this study, three common features, including correlation, dissimilarity, and energy were selected, and the calculation formulas are shown in Table 2.

Shape features can reflect the shape information of an object in a remote sensing image and describe the assemblage of its shape features, which helps to avoid the phenomena of “same object with different spectra, different objects with same spectrum” [36]. The area, length, width, shape index, and aspect ratio are generally used to describe shape features. In this study, the area, shape index, and aspect ratio were selected. The calculation formulas are shown in Table 3.

##### Feature Selection

Considering the spectral, texture, and shape features of objects and multi-band characteristics, data redundancy is inevitable. To reduce this redundancy and improve feature quality, it is necessary to select features that effectively describe the information of each object. The SDT is used to test the difference between the experimental group and the control group, or the effect of two different treatments, and whether the difference is significant or not [37]. Therefore, we used the SDT to calculate the significance difference for different land cover types amongst the selected category features in each band for feature optimization. The greater the difference in features, the more significant the band features. This also demonstrates that the features of this band can be selected as optimal features. Variance analysis was applied to perform the SDT in this study. The significant difference was calculated according to Equation (3).

(3) $$F=MS_{b}/MS_{w}$$

where,

(4) $$MS_{b}=SS_{b}/V_{b}$$

(5) $$MS_{w}=SS_{w}/V_{w}$$

(6) $$SS_{b}=\sum_{i}\frac{{\left(\sum_{j}X_{ij}\right)}^{2}}{b}-{\left(\sum_{i}\sum_{j}X_{ij}\right)}^{2}/N$$

(7) $$SS_{w}=SS-SS_{b}$$

(8) $$SS=\sum_{i}\sum_{j}X_{ij}{}^{2}-{\left(\sum_{i}\sum_{j}X_{ij}\right)}^{2}/N$$

(9) $$V_{b}=k-1$$

(10) $$V_{w}=N-k$$

where *F* is the statistic calculated by the analysis of variance, $MS_{b}$ is the variance between groups, $MS_{w}$ is the variance within group, $SS_{b}$ is the sum of squared deviations between groups, $SS_{w}$ is the sum of squared deviations within group, *SS* is the total sum of squared deviations, $X_{ij}$ is the *j*-th sample value of the *i*-th group, *N* is the total number of samples, *b* is the total number of samples of each group, $V_{b}$ is the degree of freedom between groups, $V_{w}$ is the degree of freedom within the group, and *k* is the total number of groups.

*F* Distribution is *F~F*$\left(v_{b},v_{w}\right)$ as in Reference [38]. According to the *F* threshold table, $F_{\alpha }\left(v_{b},v_{w}\right)$ can be found. Generally, the value of *α* is 0.05 or 0.01. By comparing *F* with $F_{\alpha }\left(v_{b},v_{w}\right)$, the significant difference can be found. For example, when *F* < $F_{0.05}\left(v_{b},v_{w}\right)$, the difference is not significant; when *F* ≥ $F_{0.05}\left(v_{b},v_{w}\right)$*,* the difference is significant; when *F* ≥ $F_{0.01}\left(v_{b},v_{w}\right)$, the difference is extremely significant.

#### 2.2.3. Change Vector Analysis

CVA was first proposed by Malila in 1980 [39]. It can express multiple characteristics of each object using one-dimension vectors of n bands for an image. In this study, because the dimension and magnitude of the features used were quite different, standardization [40] was performed for each feature prior to its use in change detection. The change vector contains all the change information for a given object between two images and can be expressed as Equation (11).

(11) $$∆G=H-G=\left(\begin{array}{c} x_{i}^{1}\left(t_{1}\right)-x_{i}^{1}\left(t_{2}\right)\\ \begin{array}{c} x_{i}^{2}\left(t_{1}\right)-x_{i}^{2}\left(t_{2}\right)\\ \ldots \\ x_{i}^{n}\left(t_{1}\right)-x_{i}^{n}\left(t_{2}\right) \end{array} \end{array}\right)$$

The feature vector of an object in a remote sensing image at time *t*_1_ and *t*_2_ is represented as $G={\left(x_{i}^{1}\left(t_{1}\right),x_{i}^{2}\left(t_{1}\right),\ldots ,x_{i}^{n}\left(t_{1}\right)\right)}^{T}$ and $H={\left(x_{i}^{1}\left(t_{2}\right),x_{i}^{2}\left(t_{2}\right),\ldots ,x_{i}^{n}\left(t_{2}\right)\right)}^{T}$, respectively, where *n* is the feature number and $x_{i}^{k}\left(t\right)$ represents the normalized value of the *k*-th feature of object *i* at time *t*.

The change intensity can be calculated using the Euclidean Distance as in Equation (12):

(12) $$\|∆G\|=\sqrt{\sum_{k=1}^{n}{\left(x_{i}^{k}\left(t_{1}\right)-x_{i}^{k}\left(t_{2}\right)\right)}^{2}}$$

where ‖∆G‖ characterizes all the feature differences between two remote sensing images. The larger the ‖∆G‖, the more likely the object is changed. Detection for changed and non-changed objects can be completed by setting the change threshold according to the value of the change intensity. By determining the change threshold, the change area can be determined from the change intensity map easily and accurately. 

#### 2.2.4. Correlation Coefficient Calculation

According to pattern recognition theory, multiple unrelated constraints should be used for recognition to avoid the limitations of single constraints [41]. In the traditional land cover detection method, only single change intensity is utilized to determine the change and unchanged areas, and its accuracy is not ideal. This study introduced the correlation coefficient and combined it with the change intensity to determine the change area. Based on the multi-scale segmentation results, we calculated the correlation coefficient between objects. When the object changes, the correlation coefficient is low; when the object does not change, the correlation coefficient is high. The correlation coefficient is calculated using Equation (13).

(13) $$R=\frac{\sum_{k=1}^{n}\left\{\left[x_{i}^{k}\left(t_{1}\right)-{\bar{x}}_{i}\left(t_{1}\right)\right]\cdot \left[x_{i}^{k}\left(t_{2}\right)-{\bar{x}}_{i}\left(t_{2}\right)\right]\right\}}{\sqrt{\sum_{k=1}^{n}{\left[x_{i}^{k}\left(t_{1}\right)-{\bar{x}}_{i}\left(t_{1}\right)\right]}^{2}\times \sum_{k=1}^{n}{\left[x_{i}^{k}\left(t_{2}\right)-{\bar{x}}_{i}\left(t_{2}\right)\right]}^{2}}}$$

where *n* is the number of bands, $x_{i}^{k}\left(t\right)$ represents the average gray value of all the pixels of object *i* in band *k* in the *t*-phase image, and ${\bar{x}}_{i}\left(t\right)$ represents the mean gray value of object *i* of the n bands in the *t*-phase image.

#### 2.2.5. Optimal Threshold Determination for Change Detection

According to the research of Tung Fung et al. [42], the Kappa coefficient based on a confusion matrix may be the most appropriate for determining the optimal change threshold among various indexes. Therefore, in this study, samples with changes and samples with no changes were selected. The change intensity and correlation coefficient with the maximum Kappa coefficient were selected as the optimal change thresholds. Then, the two thresholds were applied to detect the land cover changes. Finally, the change detection result image was generated. The confusion matrix [43] for the land cover change detection results is shown in Table 4. The misjudgment error, omission error, detection accuracy, overall accuracy, and the Kappa coefficient are calculated according to Equations (14)–(18).

(14) $$\mathrm{Misjudgment}\;\mathrm{error}=N_{nc}/N_{tc}$$

(15) $$\mathrm{Omission}\;\mathrm{error}=N_{cn}/N_{ct}$$

(16) $$\mathrm{Detection}\;\mathrm{accuracy}=N_{cc}/N_{ct}$$

(17) $$\mathrm{Overall}\;\mathrm{accuracy}=\left(N_{nn}+N_{cc}\right)/N$$

(18) $$k_{hat}=\frac{N\cdot \left(N_{nn}+N_{cc}\right)-\left(N_{tn}\cdot N_{nt}+N_{tc}\cdot N_{ct}\right)}{N^{2}-\left(N_{tn}\cdot N_{nt}+N_{tc}\cdot N_{ct}\right)}$$

where $N_{nn}$ represents the number of samples where the detection results are unchanged and have not changed in practice, $N_{cn}$ represents the number of changed samples incorrectly identified as unchanged samples, $N_{tn}$ is the total number of unchanged samples in the test results, $N_{nc}$ represents the number of unchanged samples incorrectly identified as changed samples, $N_{cc}$ represents the number of samples where the detection results are changed and have changed in practice, $N_{tc}$ represents the total number of changed samples in the test results, $N_{nt}$ represents the total number of unchanged samples in practice, $N_{ct}$ represents the total number of changed samples in practice, and *N* represents the total number of samples. $k_{hat}$ is the Kappa coefficient.

## 3. Results and Discussion

### 3.1. Multi-Scale Segmentation

**Figure 3:**
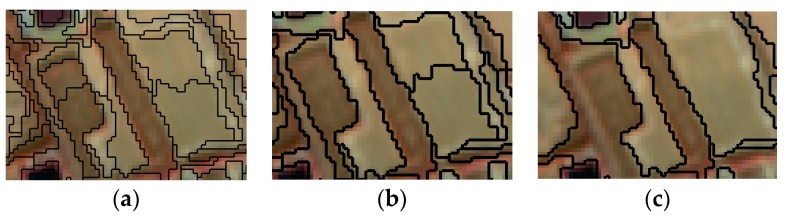
Different segmentation scale. (**a**) The weight of the shape factor is 0.2, the weight of the spectral feature is 0.8, the segmentation scale is 15. (**b**) The weight of the shape factor is 0.2, the weight of the spectral feature is 0.8, the segmentation scale is 25. (**c**) The weight of the shape factor is 0.2, the weight of the spectral feature is 0.8, the segmentation scale is 35.

**Figure 4:**
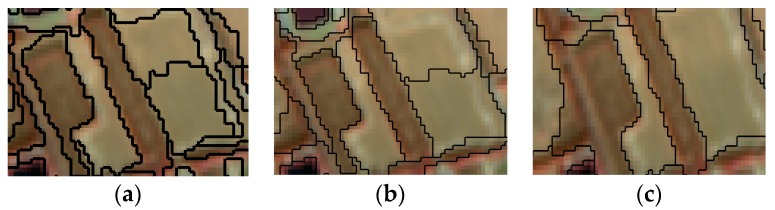
Different weights of the shape and spectral feature. (**a**) The weight of the shape factor is 0.2, the weight of the spectral feature is 0.8, the segmentation scale is 25. (**b**) The weight of the shape factor is 0.4, the weight of the spectral feature is 0.6, the segmentation scale is 25. (**c**), The weight of the shape factor is 0.6, the weight of the spectral feature is 0.4, the segmentation scale is 25.

**Figure 5:**
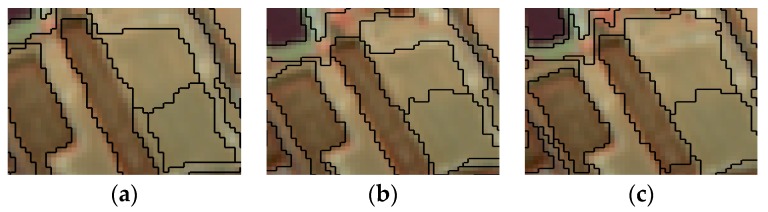
Different weights of compactness and smoothness. (**a**) The weight of smoothness is 0.5, the weight of compactness is 0.5, the segmentation scale is 25. (**b**) The weight of smoothness is 0.3, the weight of compactness is 0.7, the segmentation scale is 25. (**c**) The weight of smoothness is 0.1, the weight of compactness is 0.9, the segmentation scale is 25.

The optimal parameters for multi-scale segmentation of the remote sensing images were determined by comparison experiments. Different parameter segmentation results are shown in Figure 3, Figure 4 and Figure 5.

Figure 3 shows that when the segmentation scale is 15, the result is too fine, which makes the result complex, and when the segmentation scale is 35, the result is too rough, which will not make a clear difference. The segmentation scale was 25, which can segment the image into patches with high internal homogeneity.

Figure 4 shows that when the weights of the shape and spectral feature are 0.2 and 0.8, respectively, the result provides a clearer difference and can segment the image into patches with high internal homogeneity.

Figure 5 shows that when the weight of compactness is 0.5, the result is too fine, which makes the result complex, and when the weight of compactness is 0.9, the result is too rough, which will not make a clear difference. The weight of compactness was 0.7, which can segment the image into patches with high internal homogeneity.

**Figure 6:**
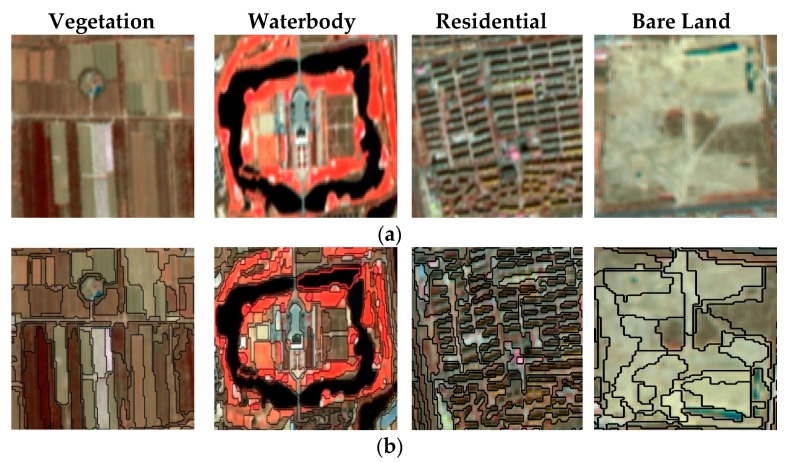
(**a**) Typical land cover types on the GF-1 images. (**b**) Multi-scale segmentation results of the GF-1 images.

Therefore, the segmentation scale was 25, the spectral and shape weights were 0.8 and 0.2, and the smoothness and compactness weights were 0.3 and 0.7, respectively. The results of the multi-scale segmentation are shown in Figure 6. The scale and weight set for the multi-scale segmentation was reasonable, since it could avoid too much fragmentation of the segmentation results, and effectively reflect the differences between the different patches, which were consistent with the feature boundaries. Therefore, the results were satisfactory.

### 3.2. Optimal Feature Selection

**Figure 7:**
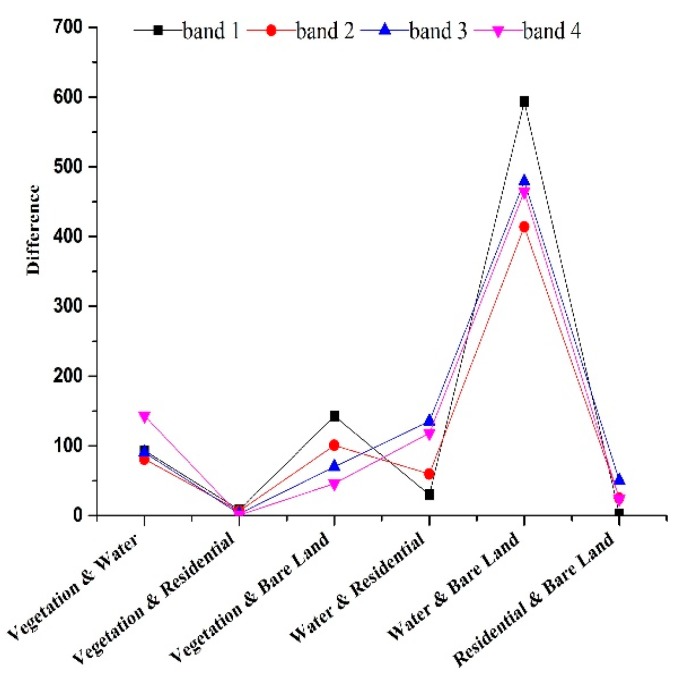
Comparison of differences in the mean grey value of each band for land cover types.

**Figure 8:**
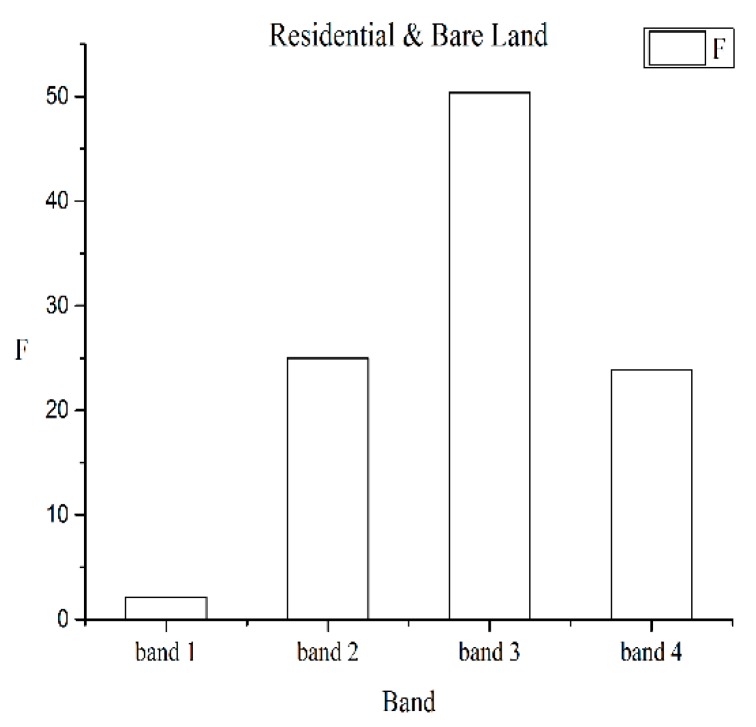
Comparison of differences in the mean grey value of each band for residential and bare land.

**Figure 9:**
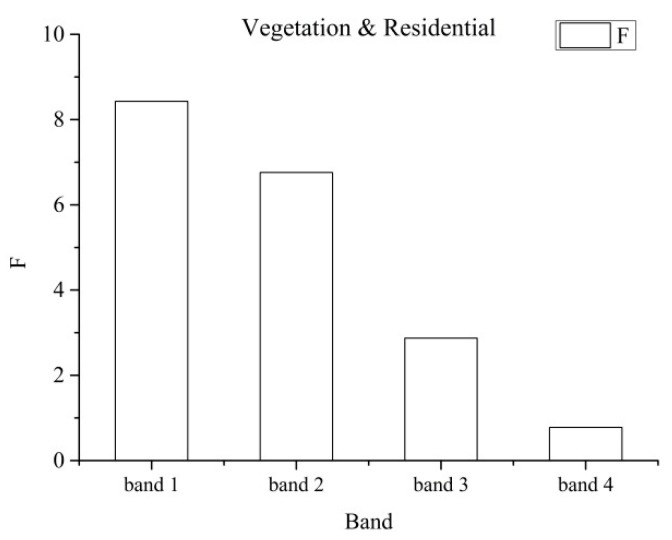
Comparison of differences in the mean grey value of each band for vegetation and residential land.

Some of the differences within features, e.g., the differences in the mean grey value and the energy of each band, are shown as follow. Figure 7 showed the differences in the mean grey value of each band. *F*_0.05_(1, 9) was the limiting value and its value was 5.1174. Bands 1, 2, 3, and 4 represented blue, green, red, and near-infrared band, respectively. Generally, when *F* was higher than *F*_0.05_(1, 9), the corresponding difference between the two categories was more significant. Compared with the differences in the mean of the spectral features (Figure 7), it showed that the differences between the residential and bare land in band 1 (Figure 8) and the differences between the vegetation and residential land in bands 3 and 4 (Figure 9), were not significant, because *F* was much lower than *F*_0.05_(1, 9). The differences between all the categories in band 2 were more significant for *F* which it was higher than *F*_0.05_(1, 9).

**Figure 10:**
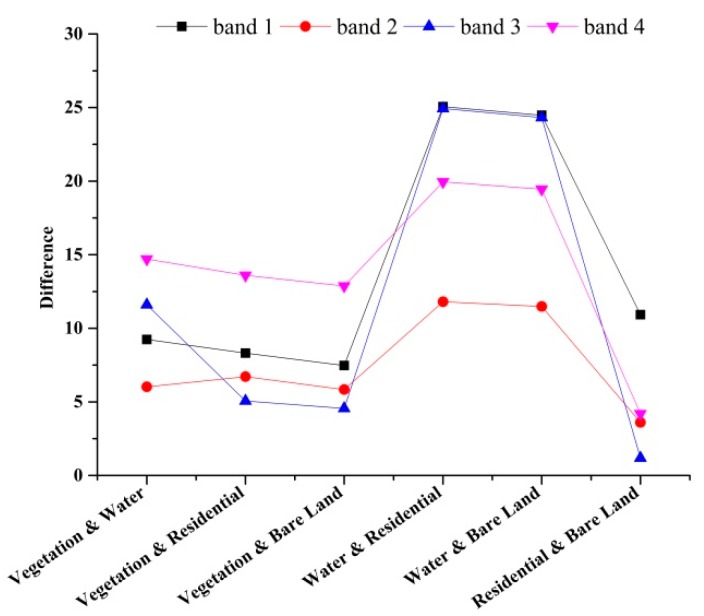
Comparison of the differences in the energy of texture feature energy differences.

Compared with the differences in the energy of the texture features (Figure 10), it showed that the differences between the residential and bare land were not significant in bands 2, 3, and 4, because the *F* was much lower than *F*_0.05_(1, 9). All the differences between the categories in band 1 were significant for *F* higher than *_F_*_0.05_(1, 9).

**Figure 11:**
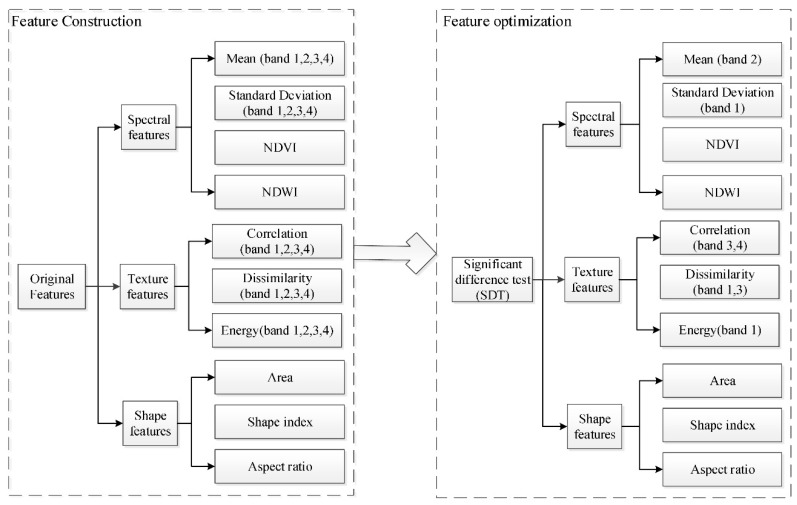
The processes of selecting the optimal features.

Using the SDT, the most distinctive features among the categories were finally selected (Figure 11). The most distinctive spectral features were the mean of band 2, the variance of band 1, the NDVI, and the NDWI. The most distinctive texture features were the correlation of band 3 and band 4, the dissimilarity of band 1 and band 3, and the energy of band 1. The shape features were the length–width ratio, area, and the shape index. In total, 12 features were selected as the optimal features for land cover change detection.

### 3.3. Change Intensity and the Correlation Coefficient

**Figure 12:**
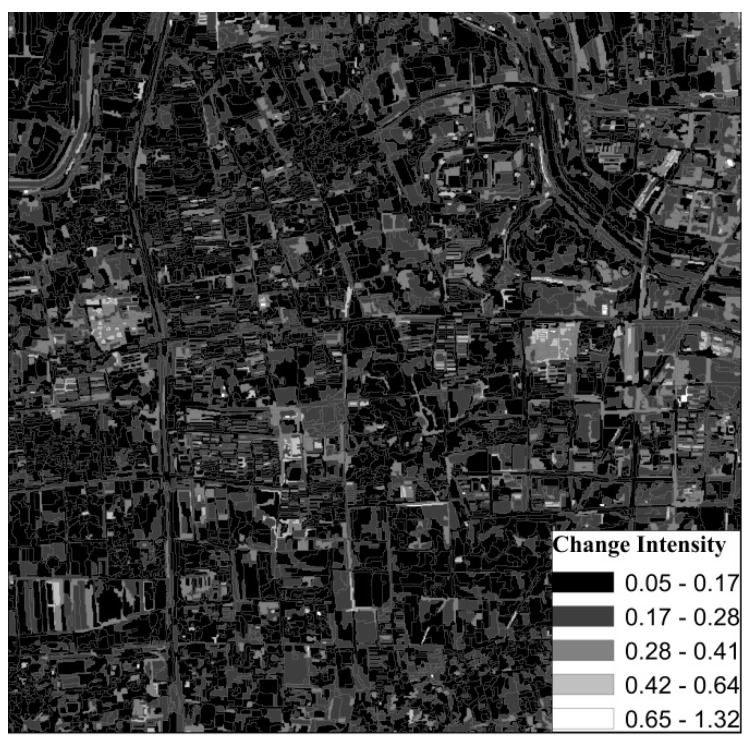
Change intensity map for the GF-1 images in 2016 and 2017.

**Figure 13:**
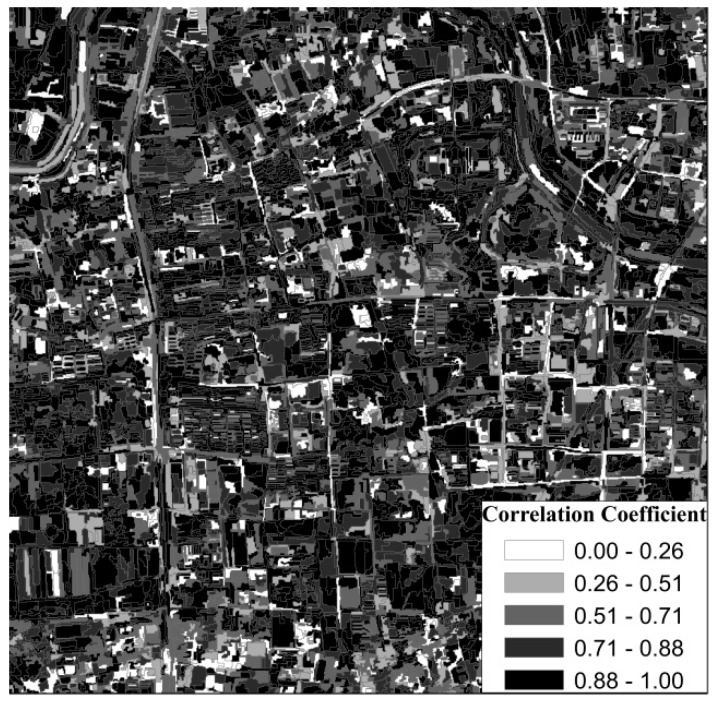
Correlation coefficient map for the GF-1 images in 2016 and 2017.

According to the multi-scale segmentation results, we calculated the change intensity and correlation coefficient of the objects. The change intensity map for the GF-1 images in 2016 and 2017 is shown in Figure 12. When the patch was changed, its change intensity value was greater and the color was brighter. When the patch was unchanged, the change intensity value was smaller and the color was darker. The correlation coefficient map for the GF-1 images in 2016 and 2017 is shown in Figure 13. When the patch was changed, its correlation coefficient was smaller and its color was brighter. When the patch was unchanged, the correlation coefficient was larger and the color was darker.

### 3.4. Land Cover Change Detection

Using visual interpretation, 213 samples were selected for model training, including 87 changed samples and 126 unchanged samples. These training samples were used to analyze the changed binary map generated during the loop computation. By calculating the Kappa coefficient, the optimal change intensity threshold and the optimal correlation coefficient threshold with the maximum Kappa coefficient were calculated. The ODCD and SCCD methods were both used for land change detection.

#### 3.4.1. Results from SCCD

**Figure 14:**
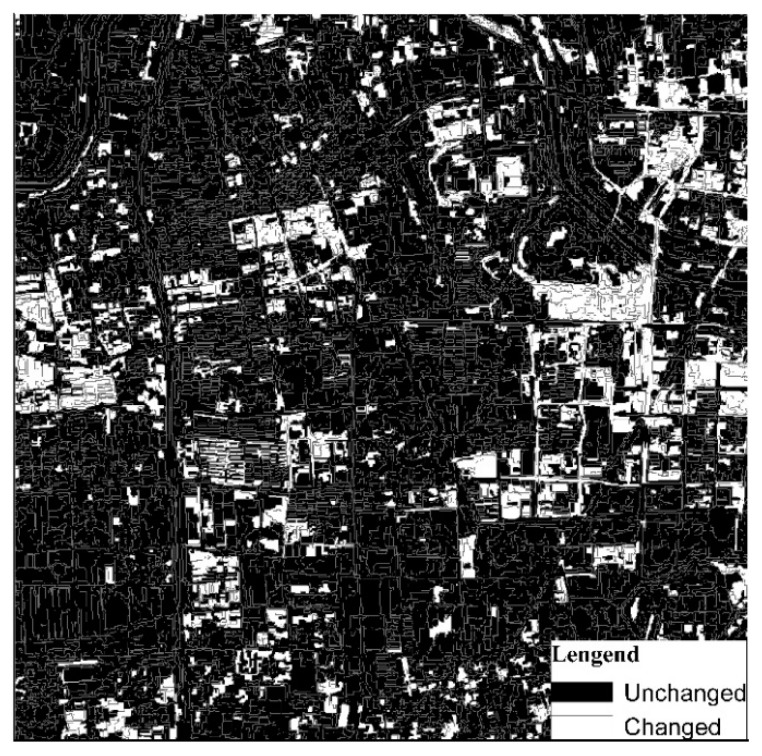
Change binary map for land cover detection based on SCCD.

**Figure 15:**
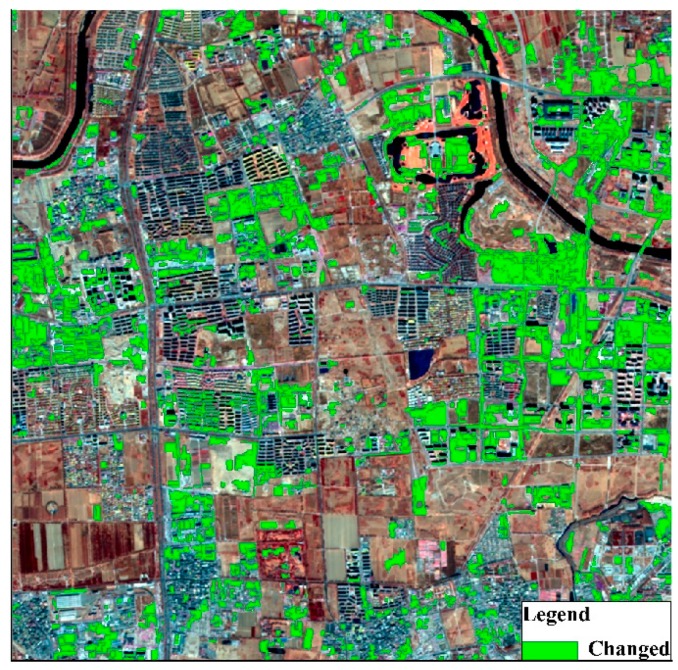
SCCD land cover change detection result overlaid on the GF-1 image of Beijing in February 2017.

When SCCD was applied for land cover change detection, only the change intensity was used. According to the training samples, the maximum value of the Kappa coefficient was 0.80 and the optimal threshold of change intensity was 0.30. That is, the object changed when the change intensity value was greater than 0.30. Based on the optimal threshold of change intensity, a final binary map was acquired as shown in Figure 14. The maximum Kappa coefficient corresponding to the confusion matrix is shown in Table 5. By overlaying the land cover change map on the GF-1 image in 2017, as shown in Figure 15, it was clear that most of the changed features involved changes from bare land and vegetation to residential, and small parts involved changes from waterbody to bare land. SCCD change detection was sensitive to seasonal changes in vegetation, spectral changes in the roofs of buildings, and spectral changes in bare land, so they are easily misclassified as changes by SCCD.

#### 3.4.2. Results from the ODCD

**Figure 16:**
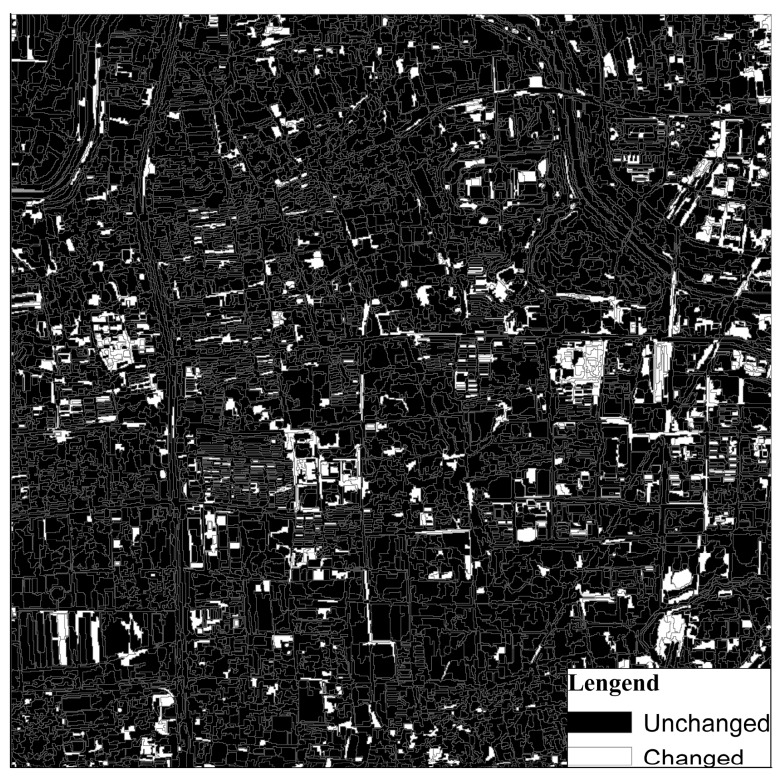
Change binary map for land cover detection based on the object-level double constrained change detection (ODCD).

**Figure 17:**
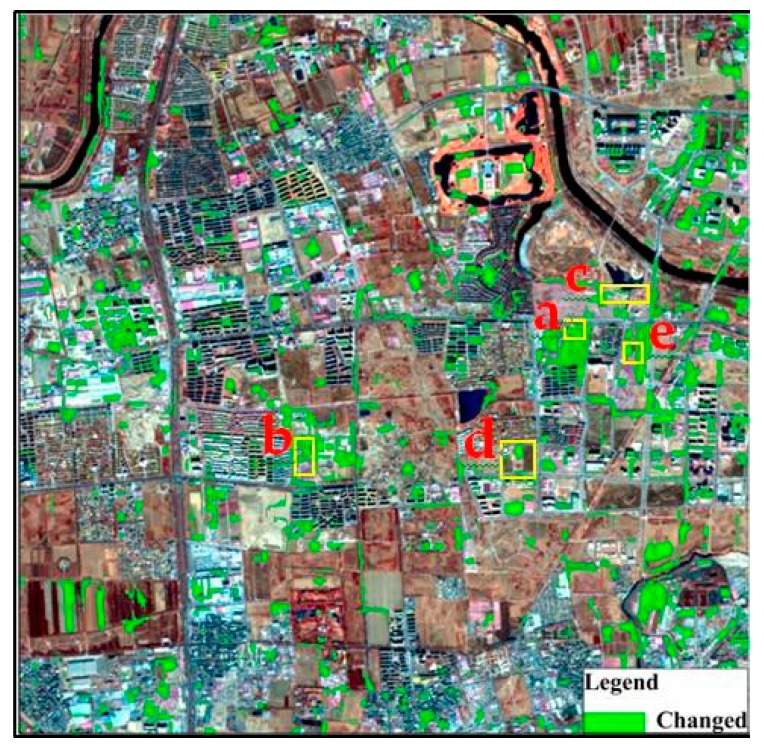
ODCD land cover change detection result overlaid on the GF-1 image of Beijing in February 2017.

**Figure 18:**
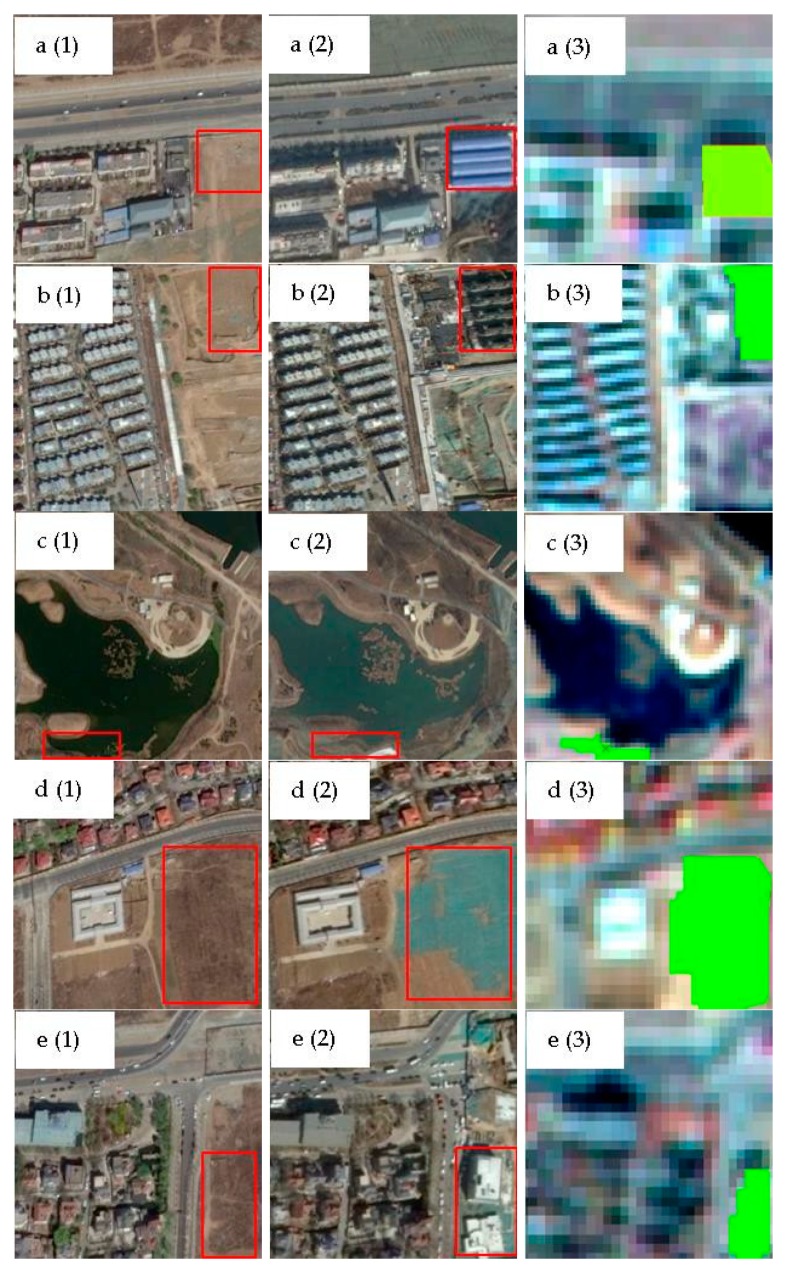
Examples of typical land cover change types: (**a(1)**,**b(1)**,**c(1)**,**d(1)**,**e(1)**): Worldview 02 Images in 2016: (**a(2)**,**b(2)**,**c(2)**,**d(2)**,**e(2)**): Worldview 02 Images in 2017: (**a(3)**,**b(3)**,**c(3)**,**d(3)**,**e(3)**): land cover change detection result using GF-1 images.

When the ODCD was applied for land cover change detection, the change intensity and correlation coefficient were both used. According to the training samples, the maximum Kappa coefficient was 0.87, and the optimal thresholds of the change intensity and correlation coefficient were 0.26 and 0.94, respectively. That is, when the change intensity was greater than 0.26 and the correlation coefficient was less than 0.94, the object changed. According to the optimal change intensity and correlation coefficient thresholds, the change binary image was acquired as shown in Figure 16. By overlaying the land cover change map on the GF-1 image in 2017 as shown in Figure 17, some typical change examples are shown in Figure 18. The maximum Kappa coefficient corresponding to the confusion matrix is shown in Table 6, and it is clear that the overall accuracy and the Kappa coefficient of the ODCD are higher than that of the SCCD. The ODCD can make up for the shortcomings in the seasonal sensitivity of SCCD and improve the accuracy of change detection.

Among all the above examples, the ODCD efficiently detected the changed areas. The typical change detection results in Figure 18a,b show changes from bare land to residential, and the results in Figure 18c show changes from waterbody to bare land, whilst the results in Figure 18d show changes from vegetation to bare land. The results in Figure 18e show changes from vegetation to residential. Owing to the resolution of the images, some small details were detected as changed areas, such as shadows between buildings.

### 3.5. Precision Comparison

To further confirm the validity and accuracy of the ODCD, a total of 333 samples, excluding the samples used to determine the optimal change threshold, were selected for validation, including 133 changed samples and 200 unchanged samples. For the analysis of the change detection results, we compared the results of the ODCD and SCCD. The overall accuracy, Kappa coefficient, and overall error were selected as the accuracy evaluation indexes. The quantitative evaluation results are shown in Table 7 and Table 8. The accuracy comparison between the ODCD and SCCD is shown in Table 9. The overall accuracy and the Kappa coefficient of the ODCD were higher than that of SCCD. For overall accuracy, the overall accuracy of the ODCD was 92.19% while the overall accuracy of SCCD was 81.98%. The overall accuracy of the ODCD was about 10% higher than that of SCCD. For the Kappa coefficient, the Kappa coefficient of the ODCD was 0.84 and the Kappa coefficient of SCCD was 0.62. The Kappa coefficient of the ODCD was about 0.22 higher than that of SCCD. For the error, misjudgment error, and the omission error, the results for the ODCD were lower than the results of SCCD. The misjudgment error was 15% lower and the omission error was 12% lower. The total error of the ODCD was 27% lower than that of SCCD.

For testing whether the differences of accuracy were statistically significant, we carried out a hypothesis statistical test (*t*-test) to confirm that the differences in accuracy were statistically significant. An additional total of 121 samples were selected for validation, including 89 changed samples and 32 unchanged samples. The quantitative evaluation results are shown in Table 10, Table 11 and Table 12. The t-test was used to test whether the differences were statistically significant. *α* is the limiting value in the *t*-test, and its value is generally 0.05. When the *p*-value calculated by the *t*-test was less than *α*, it could be concluded that the differences were statistically significant [44]. The results are shown in Table 13.

Given the *p*-value was less than 0.05, the differences were statistically significant. The validity and accuracy of the ODCD could be confirmed.

## 4. Conclusions

The ODCD proposed for land cover change detection in this study resolved the problem of computational complexity due to excessive feature variables in feature extraction. However, the problem of using empirical judgment to determine the change threshold makes the results less objective and less accurate. The ODCD employs a more objective change threshold determination algorithm that achieves simultaneous determination of the thresholds of the change intensity and the correlation coefficient based on maximizing the Kappa coefficient. It is a more effective method for land cover change detection. The major conclusions are as follows.

(1) Combining change vector analysis with correlation coefficients based on object-level, the ODCD can reduce the shortcomings of seasonal sensitivity of SCCD and improve the accuracy of land cover change detection. The ODCD’s overall accuracy was 92.19% and this was 10% higher than that of SCCD. At the same time, its overall error was 20% and it was 27% lower than that of SCCD.
(2) ODCD can be used to reduce the number of features and improve the computational efficiency. The SDT is an effective feature optimization method. Using optimal feature selection, the feature dimensions were reduced from 26 to 12, which increased the calculation speed.

Therefore, the ODCD can provide a useful reference for ecological environment assessment and land use planning. At the same time, the ODCD can provide an effective reference for remote sensing in land cover change detection.

Future studies on the ODCD should involve a greater number of different objects, such as objects related to residential change detection. In addition, the image resolution in this study led to some areas being detected as changed areas; therefore, further studies should also utilize different resolution images. 

## Figures and Tables

**Table 1:** Spectral features.

Feature Parameters	Formula	Formula Description
Mean	$\mu =\frac{1}{n}\overset{n}{\underset{i=1}{\sum }}V_{i}$	μ is the sum of all pixel values $V_{i}$ divided by the total number of pixels in one object.
Standard deviation	$\delta =\sqrt{\frac{1}{n-1}\overset{n}{\underset{i=1}{\sum }}{\left(V_{i}-\mu \right)}^{2}}$	$V_{i}$ is the value of all pixels in the object, μ is the mean of the object.
NDVI	$\mathrm{NDVI}=\frac{\rho_{NIR}-\rho_{R}}{\rho_{NIR}+\rho_{R}}$	$\rho_{NIR}$ is the reflectance for the near-infrared band, $\rho_{R}$ is the reflectance for the infrared band.
NDWI	$\mathrm{NDWI}=\frac{\rho_{Green}-\rho_{NIR}}{\rho_{Green}+\rho_{NIR}}$	$\rho_{Green}$ is the reflectance of the green band, and $\rho_{NIR}$ is the reflectance of the near-infrared band.

**Table 2:** Texture features.

Features Parameters	Formula	Formula Description
Correlation	$\mathrm{Correlation}=\overset{k}{\underset{i=1}{\sum }}\overset{k}{\underset{j=1}{\sum }}\frac{\left(ij\right)P\left(i,j\right)-u_{i}u_{j}}{S_{i}S_{j}}$	*i* is the gray value of any point in the image; *j* is the gray value of another point deviating from the point; P(i,j) is the frequency of occurrence of the gray pair in the gray level co-occurrence matrix, $u_{i}$ and $u_{j}$ represent the mean values in the row and column direction, respectively, and $S_{i}$ and $S_{j}$ represent the variance in the row and column direction, respectively. It reflects the consistency of image texture and the degree of similarity of metric co-occurrence matrix elements in the row or column direction.
Dissimilarity	$\mathrm{Dissimilarity}=\overset{n-1}{\underset{i,j=0}{\sum }}P\left(i,j\right)\left|i-j\right|$	P(i,j) is the frequency of occurrence of the gray pair in the gray level co-occurrence matrix. The higher the local contrast, the higher the similarity.
Energy	$\mathrm{Energy}=\overset{n-1}{\underset{i,j=0}{\sum }}P{\left(i,j\right)}^{2}$	P(i,j) is the frequency of occurrence of the gray pair in the gray level co-occurrence matrix. Energy is also called “the angle second moment.” When the image is a homogeneous area with a consistent texture, its energy is greater.

**Table 3:** Shape features.

Feature Parameters	Formula	Formula Description
Area	$A=\overset{n}{\underset{i=1}{\sum }}x_{i}$	$x_{i}$ is the value of pixel *i*. This describes the size of the object. For non-geographically referenced data, the area of the pixel is 1.
Aspect ratio	$\gamma =\frac{l}{w}=\frac{eig_{1}\left(s\right)}{eig_{2}\left(s\right)}$	*S* is the covariance matrix composed of the coordinates of points after object vectorization, *w* is the width, and l is the length of each object.
Shape index	$\mathrm{SI}=\frac{p}{4\times \sqrt{A}}$	The variable *p* is the perimeter of the image object, *A* is the area of the image object. This describes the compactness of an object. The higher the compactness, the greater the density, and the more similar the shape is to a square.

**Table 4:** Confusion matrix for land cover change detection.

		Assessment Data		
		Unchanged	Changed	Total
Test results	Unchanged	$N_{nn}$	$N_{cn}$	$N_{tn}$
	Changed	$N_{nc}$	$N_{cc}$	$N_{tc}$
	Total	$N_{nt}$	$N_{ct}$	N

**Table 5:** Confusion matrix for the maximum Kappa coefficient of single-constrained change detection (SCCD).

		Verification Samples		Total	User Accuracy (%)
		Unchanged	Changed		
Test Results	Unchanged	121	5	126	96.03
	Changed	15	72	87	82.70
	Total	136	77	213	
Producer Accuracy (%)		88.97	93.50		

Overall accuracy = 90.61%; Kappa coefficient = 0.80.

**Table 6:** Confusion matrix for the maximum Kappa coefficient of the ODCD.

		Verification Samples		Total	User Accuracy (%)
		Unchanged	Changed		
Test Results	Unchanged	122	8	130	93.85
	Changed	5	78	83	93.98
	Total	127	86	213	
Producer Accuracy (%)		96.06	90.70		

Overall accuracy = 93.9%; Kappa coefficient = 0.87.

**Table 7:** Confusion matrix of SCCD.

		Verification Samples		Total	User Accuracy (%)
		Unchanged	Changed		
Test Results	Unchanged	172	28	200	86.00
	Changed	32	101	133	75.94
	Total	204	129	333	
Producer Accuracy (%)		84.31	78.29		

**Table 8:** Confusion matrix of ODCD.

		Verification Samples		Total	User Accuracy (%)
		Unchanged	Changed		
Results	Unchanged	186	14	200	93.00
	Changed	12	121	133	90.98
	Total	198	135	333	
Producer Accuracy (%)		93.94	89.63		

**Table 9:** Accuracy comparison table between SCCD and ODCD.

	Overall Accuracy	Kappa Coefficient	Total Error	
			Misjudgment Error	Omission Error
SCCD	81.98%	0.62	24%	22%
ODCD	92.19%	0.84	9%	10%

**Table 10:** Confusion matrix of SCCD.

	Unchanged	Changed	Total
Unchanged	73	16	89
Changed	4	28	32
Total	77	44	121

**Table 11:** Confusion matrix of the ODCD.

	Unchanged	Changed	Total
Unchanged	83	6	89
Changed	4	28	32
Total	87	34	121

**Table 12:** Accuracy comparison table between SCCD and ODCD.

	Overall Accuracy	Kappa Coefficient
SCCD	83.66%	0.62
ODCD	91.73%	0.80

**Table 13:** *t*-Test for difference.

	Overall Accuracy Difference	Kappa Coefficient Difference
Training group	3.29%	0.07
Verification group 1	10.21%	0.18
Verification group 2	8.07%	0.22
*p* value	0.03186	0.02286
